# Neurological and neurobehavioral assessment of experimental subarachnoid hemorrhage

**DOI:** 10.1186/1471-2202-10-103

**Published:** 2009-08-25

**Authors:** Hyojin Jeon, Jinglu Ai, Mohamed Sabri, Asma Tariq, Xueyuan Shang, Gang Chen, R Loch Macdonald

**Affiliations:** 1 Division of Neurosurgery, St. Michael's Hospital, Keenan Research Centre in the Li Ka Shing Knowledge Institute of St. Michael's Hospital and Department of Surgery, University of Toronto, Toronto, Ontario, Canada

## Abstract

About 50% of humans with aneurysmal subarachnoid hemorrhage (SAH) die and many survivors have neurological and neurobehavioral dysfunction. Animal studies usually focused on cerebral vasospasm and sometimes neuronal injury. The difference in endpoints may contribute to lack of translation of treatments effective in animals to humans. We reviewed prior animal studies of SAH to determine what neurological and neurobehavioral endpoints had been used, whether they differentiated between appropriate controls and animals with SAH, whether treatment effects were reported and whether they correlated with vasospasm. Only a few studies in rats examined learning and memory. It is concluded that more studies are needed to fully characterize neurobehavioral performance in animals with SAH and assess effects of treatment.

## Introduction

Mortality and morbidity from aneurysmal subarachnoid hemorrhage (SAH) have decreased with improvements in surgery, pharmacological treatment and intensive care. The overall outcome, however, remains relatively poor [1,2]. Management of SAH includes early obliteration of the ruptured aneurysm to prevent rebleeding, prevention of secondary brain injury from such things as decreased cerebral perfusion and prevention and treatment of delayed neurological deterioration secondary to cerebral vasospasm. The case fatality rate is approximately 50% and 30% of survivors remain dependent on others, mainly due to the persistent cognitive impairment rather than focal neurological deficits [3].

**Figure 1:**
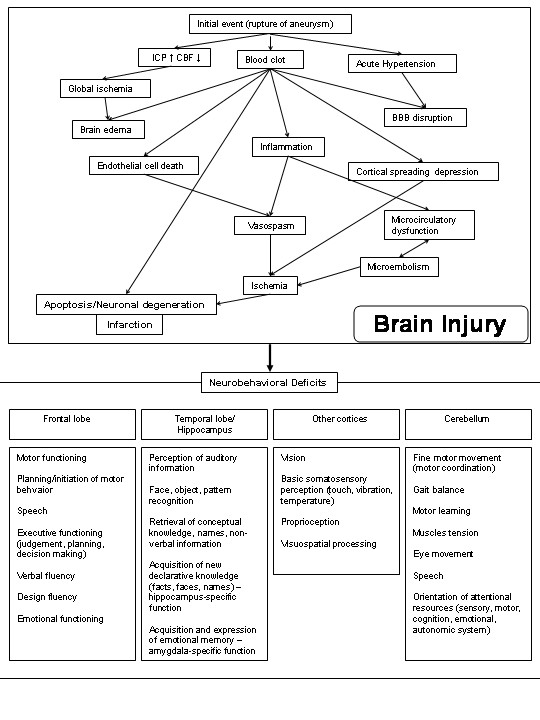
**The pathophysiology of brain injury after SAH may originate from 3 phenomena; transient global ischemia (due to increased intracranial pressure and decreased cerebral perfusion pressure), subarachnoid blood clot and acute hypertension**. These may lead to a variety of secondary effects including brain edema, delayed large artery vasospasm, breakdown of the BBB, microcirculatory changes, thromboemboli, cortical spreading depression and delayed neuronal death due to apoptosis or other mechanisms. The end result is focal and scattered brain injury. The role of astrocytes is increasingly being recognized also. In the end, these processes have to cause neurological and neurobehavior deficits to be important and these will depend on what areas of the brain or networks in the brain are disrupted.

Although the mechanisms underlying the cognitive deficits have not been well studied, they have nevertheless been attributed to ischemic brain injury occurring either during the initial hemorrhage or as a consequence of macro- and microvascular dysfunction and delayed ischemic neurological deterioration (Figure 1) [1]. Other mechanisms, including delayed neuronal death and cortical spreading depression have been suggested [4,5]. These processes may lead to large-artery territory infarction, smaller cortical laminar infarcts or possibly other types of selective neuronal death or perhaps even dysfunction in the absence of detectable death [6].

Much work on SAH has focused on cerebral vasospasm. This is based on the assumption that severe vasospasm can reduce cerebral blood flow, cause brain ischemia and infarction and contribute to poor outcome [7]. For such studies, an acceptable dependent variable would be angiographic arterial diameter. This might not detect treatment toxicity, however. Considering the fact that the other proposed mechanisms do not necessarily cause focal cerebral infarctions, how to assess outcome is a problem. Clinically, neurobehavioral testing could be used and generally is done 3 to 6 months post-SAH.

Animal studies have often relied on histological assessment of neuron death but there are several problems with this. The time course of changes needs to be considered since complications of SAH are often delayed for several days. Not much is known about the time course of neuronal injury after SAH but it is notable that neuron death seems to progress over months after experimental ischemic stroke [8]. Furthermore, lack of neuron death associated with a treatment does not necessarily indicate that the rescued neurons are functional. Studies show ischemia treated with ischemic preconditioning or hypothermia prevents neuron death but that behavior is not improved and/or there is an inability to induce long term potentiation in hippocampal slices [8,9]. Therefore, it seems warranted to employ neurobehavioral testing in models of SAH.

In this paper, we hypothesize that SAH models in animals should cause neurological and neurobehavioral alterations that do not occur in sham-operated animals. Treatments that improve histological or other measures of brain injury should also improve the neurological/neurobehavioral alterations. To this end, literature studying neurological and neurobehavioral alterations after experimental SAH is reviewed to determine what has been done, whether tests used thus far differentiated between appropriate controls and animals with SAH, whether treatment effects were reported and whether neurobehavioral tests correlated with vasospasm. Prior review of animal models focused on vasospasm and didn't mention these endpoints [10]. This review is not exhaustive and we apologize for any omissions. The purpose is not to review the pathophysiology of brain injury after SAH, although when relevant, some discussion of this is provided.

## Rats

### Models

The most common methods of inducing SAH in rats are to inject blood into the cisterna magna once (single injection) or twice (separated by 1 or 2 days, double injection) or to perforate an anterior circulation intracranial artery endovascularly (perforation model)[10]. Prunell, et al., developed an anterior circulation single injection model where blood was injected into the chiasmatic cistern [11].

### Endpoints Used

#### Mortality

Mortality tends to be lowest with the single injection, is higher with the double-injection and highest with the endovascular perforation model (Table 1) [12-23]. Mortality is probably lower if sham surgery is done with injection of artificial cerebrospinal fluid (CSF) or physiological saline but this has seldom been documented. Intracranial pressure also is not usually measured so it is difficult to differentiate effects of subarachnoid blood from those of increased intracranial pressure. The larger the injection volume, the higher the mortality. High mortality rates can be problematic because this may remove animals that have neurologic deficits, leaving only relatively normal animals for assessment.

**Table 1:** Selected Studies of SAH in Rats Examining Mortality and Neurological Endpoints

**Author**	**Model**	**Mortality**	**Behavior Tests**	**Controls**	**Experimental findings**	**Disconnect between vasospasm and outcomes**
Davella1990A	Single 300 μl injection into cisterna magnia within 10-15 seconds, ICP not monitored	34/200 (17%)	No specific scales, observed rats for neurological dysfunction, drinking and feeding and body weight	SAH (n = 200), saline-injected (n = 100) controls or untreated controls (n = 60)	No acute or delayed neurological dysfunction, >90% of surviving rats resumed normal activity within 3 days, 2.6% reduction in body weight 36 hours after SAH but eating/feeding returned to normal within 3 days. CSF levels of eicosanoids (PGE2, PGF2a, TXB2) were significantly higher after SAH compared to noninjected and mock-CSF injected rats. The increase in eicosanoids was accompanied by a decrease in the mean vascular diameter (78~82% of control) 2 days after cisternal injection	They correlated
Germano1994	Single 400 μl injection into cisterna magna within 15-20 seconds, no ICP monitoring	None reported	Duration of suppression of simple nonpostural (pinna reflex, corneal reflexes, startle response) and complex postural somatomotor function (righting response, spontaneous locomotion, escape response) after SAH. Beam balance, beam walking tests and body weight for 5 days after SAH	SAH (n = 10), saline-injected (n = 10) and sham-operated (n = 10) controls	No acute neurological deficits or difference between SAH or saline-injected controls, significant deficits seen with SAH rats on beam balance 1 day after, beam walking test 1-4 days and body weight 1-5 days after SAH	Not assessed
Germano1998C	Single 400 μl injection into cisterna magna within 30 seconds, no ICP monitoring	None reported	beam balance, beam walking, body weight	SAH or sham-operated controls	SAH associated with impaired beam balance and walking and decreased body weight compared to sham, AVS improved neurological function, preserved the blood-brain barrier and decreased vasospasm	They correlated
Imperatore2000	Single 400 μl injection into cisterna magna within 30 seconds, no ICP monitoring	None reported	Beam balance, beam walking	SAH or sham-operated controls	SAH associated with impaired beam balance and walking and decreased body weight compared to sham, AVS preserved the blood-brain barrier at 48 hours and improved behavior, no assessment of vasospasm	Not assessed
Carpenter2001	2 injections of 250-300 μl into cisterna magna, no ICP monitoring	3/80 (3%)	General observations	SAH or saline injected controls	All animals drowsy the day after injection but then recovered, SAH associated with changes in purinergic receptors in the basilar artery	Not assessed
Germano2002	Single 400 μl injection into cisterna magna within 30 seconds, no ICP monitoring	None reported	Beam balance, beam walking, body weight	SAH or sham-operated controls	SAH associated with impaired beam balance and walking and decreased body weight compared to sham, calpain inhibitor decreased deficits and improved blood-brain barrier integrity at 48 hours	Not assessed
Prunell2002	Single injection 200 - 300 μl into chiasmatic cistern, ICP monitoring	25% with 200, 50% with 250 and 100% with 300 μl	None	SAH or saline-injected controls	ICP, amount of SAH were measured	Not assessed
Gules2002	Single or double injections into cisterna magna or endovascular perforation	0% single hemorrhage, 9% double hemorrhage, 57% endovascular perforation	None	None	Mortality highest with endovascular perforation model	Not assessed
Prunell2003	Chiasmatic injection 200 μl, cisterna magna injection or endovascular perforation, with ICP monitoring	44% endovascular, 25% chiasmatic, 0% cisterna magna injection	None	None	Mortality highest with endovascular perforation model	Not assessed
Zausinger2004	Endovascular perforation	65% in control saline, 60% with 7.5% NaCl and 35% with 7.5% NaCl and dextran	100 point neurological score composed of general behavior and respiration (40), cranial nerves (20), sensitivity to tactile stimuli (10), motor (10), coordination (20) {Katz1995}	None	Significantly better neurological scores within first days of SAH and less neuronal death at 7 days after 7.5% NaCl plus dextran treatment, trend towards better weight and lower mortality	Not assessed
Park2004	Endovascular perforation	11/26 (42%) of SAH rats died at 24 hours, statistically insignificant decrease from 43% with sham DMSO to 25% of z-VAD-FMK group	Modified 25-point scale testing neurological function {Garcia1995}	SAH or sham-operated controls	SAH reduced significantly the neurological score at 6 and 24 hours, pancaspase inhibitor z-VAD-FMK decreased TUNEL and caspase 3 in endothelial cells, decreased caspase 3 activation, reduced blood-brain barrier permeability, decreased vasospasm and brain edema and improved neurological outcome	They correlated
Ostrowski2005	Endovascular perforation	20/42 (48%)	Modified 25-point scale testing neurological function {Garcia1995}	SAH or sham-operated controls	SAH reduced neurological function significantly 24 hours after SAH, hyperbaric oxygen marked reduced mortality and also decreased ICP, improved CBF, slightly improved brain edema and neuronal death, decreased TUNEL staining in hippocampus	Not assessed
Prunell2005	Endovascular perforation or prechiasmatic SAH	46% endovascular, 24% prechiasmatic	None	SAH or sham-operated controls	SAH associated with TUNEL positive cells, no vasospasm, reduced CBF did occur	No vasospasm yet TUNEL positive neurons and decreased CBF
Cahill2006	Endovascular perforation	35/140 (33%)	Modified 25-point scale testing neurological function {Garcia1995}	SAH or sham-operated controls	Increased mortality and poorer neurological scores after SAH than sham surgery, pifithrin α associated with better neurological scores, less brain edema and blood-brain barrier breakdown, less vasospasm, less basilar artery apoptosis	They correlated
Bermueller2006	Endovascular perforation	60% SAH, 40% saline, 73% saline + dextran, 73% mannitol	91 point neurological score composed of general behavior and respiration (40), cranial nerves (16), sensitivity to tactile stimuli (10), motor (10), coordination (15) {Katz1995}, 6 grade neuroscore {Bederson1986} and prehensile traction test {Zausinger2000}	None	Better behavior with NaCl 7.5% + dextran 70 6%, less brain damage, lower ICP than after treatment with NaCl or mannitol	Not assessed
Cahill2007	Endovascular perforation	35% of 195	Modified 25-point scale testing neurological function {Garcia1995}	SAH or sham-operated controls	Pifithrin α decreased mortality, improved behavior and decreased blood-brain barrier disruption, brain edema, p53, cytochrome C, TUNEL staining and neuron injury	No vasospasm measurements but neuronal damage in areas not thought to be supplied by arteries that develop vasospasm in this model
Germano2007	Single 400 μl injection into cisterna magna within 30 seconds, no ICP monitoring	None reported	Beam balance, beam walking, body weight	SAH or sham-operated controls	SAH associated with significant deficits in beam balance scores on days 1 and 2 and in beam balance times days 1-3. SAH also increased latency to cross beam days 1-4. Body weight decreased days 1-5. Felbamate improved behavior scores, body weight and decreased blood-brain barrier disruption	Not assessed
Scholler2007	Endovascular perforation	32% at 72 hours	175 point neurological score composed of general behavior and respiration (40), cranial nerves (20), sensitivity to tactile stimuli (50), motor (50), coordination (15) {Katz1995}	SAH or sham-operated controls	Significant deficits 6 and 24 hours after SAH, less by 72 hours in surviving animals compared to sham-operated. SAH associated with blood-brain barrier disruption as evidenced by albumin leakage into brain, fewer microvessels and disrupted collagen 4 all on the side of the SAH	Not assessed
Yatsushige2007	Endovascular perforation	0% sham, 35% SAH at 24 hours, 23% with treatment with SP600125	16 point score that graded mobility, reflexes, behavior and beam walking tests {Feldman1996}	SAH or sham-operated controls	SAH associated with significant behavior abnormalities, SP600125 decreased neuronal injury by decreasing caspase-3 activation and deoxyribonucleic acid damage, decreased aquaporin 1 upregulation and brain water, reduced MMP 9 activation and collagen 4 degradation, prevented blood-brain barrier disruption and a trend towards reduced mortality and better neurological function	Not assessed
Thal2008	Endovascular perforation	13/20 SAH (65%), 12/20 hypertonic saline group (60%), 7/20 hypertonic saline + dextran (35%)	Beam balance, prehensile traction, rotarod, 6 point score {Bederson1995} which is actually {Bederson1986A}, 100 point neuroscore general behavior and respiration (40), cranial nerves (20), sensitivity to tactile stimuli (10), motor (10), coordination (20) {Katz1995}	None	No significant differences among groups except on 100 point neuroscore on which hypertonic saline + dextran group had significantly less neurological deficit on day 1 as compared to other groups	Not assessed
Takata2008	Cisterna magna injection 0.5 ml over 10 minutes, 0.3 ml 2 days later, shams had saline injection, ICP not monitored	None reported	Longterm sensorimotor and cognitive function, cerebrovascular diameter and microangiography, 8-hydroxy-2-deoxyguanosine immunohistocchemistry, regional CBF	SAH or saline-injected controls	Deficits in rotarod, vertical screen and balance beam, Morris water maze detected deficits in visual spatial memory, decreased CBF for days, minimal proximal large artery vasospasm (at 3 days), significant neuronal loss in CA1 hippocampus associated with microvascular filling defects	Vasospasm was minimal but many other deficits were noted, authors suggested changes were not due to increased ICP because they injected slowly and the blood pressure increased so cerebral perfusion pressure was thought to be adequate
Silasi2008	Endovascular perforation	33%	Tapered beam, limb use asymmetry, horizontal ladder tasks, Morris water maze	SAH or sham-operated controls	SAH not associated with deficits in tapered beam, limb use asymmetry, horizontal ladder tasks, SAH did cause deficits in Morris water maze when they had to learn new location of the platform (longer mean latency and distance swum to find platform)	Not assessed
Sugawara2008	Endovascular perforation	0% sham, 16% SAH, 4% simvastatin	SAH grading (0-3 in 6 cisterns), neurological assessment (0-3 points on spontaneous movement, spont movement of all limbs, movement of forelimbs while being held by tail, climbing in cage, reaction to touch on both sides of trunk, response to vibrissae touch) {Garcia1995}	SAH or sham-operated controls	SAH associated with neurological deficits, simvastatin prevents vasospasm and improved neurological grade	Correlation between SAH grade, vasospasm and neurological score
Sugawara2008A	Endovascular perforation	0% for sham-operated, varied with SAH from 4-35%, lowest with high-dose simvastatin but none of SAH groups significantly different	SAH grading (0-3 in 6 cisterns), neurological assessment (0-3 points on spontaneous movement, spont movement of all limbs, movement of forelimbs while being held by tail, climbing in cage, reaction to touch on both sides of trunk, response to vibrissae touch) {Garcia1995}	SAH or sham-operated controls	SAH or SAH with low-dose simvastatin associated with neurological deficits, these were prevented by high dose simvastatin, the phosphatidylinositol 3-kinase inhibitor wortmannin antagonized effects of simvastatin	Correlation between vasospasm and neurological score
Gao2008A	Endovascular perforation	44% (7/16) with SAH and placebo treatment died, 38% (6/16) with SAH + tetramethylpyrazine died, none of the sham-operated controls died, not significantly decreased by tetramethylpyrazine	Modified 25-point scale testing neurological function {Garcia1995}	SAH or sham-operated controls	SAH associated with behavior deficicts, tetramethylpyrazine improved behavior at 24 hours compared to SAH, as well as brain water content, Evans blue leakage, vasospasm and decreased apoptosis markers	They correlated

#### Body Weight

Body weight reflects in part feeding and drinking behaviour and can be used to assess appetite and motivation. Body weight decreases significantly after SAH created by cisternal blood injection in rats but tends to return to normal within 3 to 5 days [1,12,13,24,25]. Injecting 300:l blood is associated with less change in body weight than after injecting 400:l. Injection times also were different (15 seconds for 300:l and 30 seconds for 400:l). The injection time affects how high the intracranial pressure rises during the injection and could also affect body weight and neurologic function by causing additional injury beyond that due to SAH itself. Indeed, injection of saline into the cisterna magna of rats also can be associated with weight loss [13].

#### General Neurological Function

Germano and colleagues provided perhaps the first more detailed neurological evaluation of rats undergoing injection of artificial CSF, autologous blood or nothing into the cisterna magna [13]. This comprised simple nonpostural somatomotor functions (duration of suppression of the pinna reflex, corneal reflexes, startle response) and acute complex postural somatomotor functions (righting response, spontaneous locomotion, escape response) that were summarized from tests developed by other investigators. Detailed quantification was not done and there were no differences between rats undergoing sham-operation or SAH created by cisternal blood injection.

Zausinger, et al., modified a scale developed for assessing neurological function after asphyxia cardiac arrest in rats (Table 2)[19,26]. The scale was adapted from one developed to study cardiac arrest in dogs [27]. Animals with endovascular SAH had impaired scores by 7 days after SAH in 3 studies but there was no comparison to sham-operated controsls so whether the score detects effects of SAH was not determined [19,28,29]. A variation of this scale with 175 points did compare sham operated to rats undergoing endovascular SAH [21]. Significant differences were noted between the sham and SAH groups 6 and 24 but not 72 hours after SAH.

**Table 2:** Behavior Score for Rats with SAH Adapted From Katz, et al.{Katz1995}

**Scale**			**Points**
General behavioral deficit			
	Consciousness	Explore spontaneously	0
		No attempt (comatose)	20
	Respirations	Normal	0
		Abnormal	20
Cranial nerve reflexes	Olfactory (sniffing food)	Present	0
		Absent	4
	Vision (follows hand)	Present	0
		Absent	4
	Corneal reflex	Present	0
		Absent	4
	Whisker (movement)	Present	0
		Absent	4
	Hearing (turning to clapped hands)	Present	0
		Absent	4
Motor deficit	Legs/tail movement	Normal	0
		Stiff	5
		Paralyzed	10
Sensory deficit	Legs/tail (on pinching)	Present	0
		Absent	10
Coordination	Beam walking (1.5 cm)	Present	0
		Absent	5
	Placing test	Present	0
		Absent	5
	Righting reflex	Present	0
		Absent	5
	Stopping at edge of table	Present	0
		Absent	5
Total			100

Park, et al., modified a scale that was developed to assess neurological function after focal cerebral ischemia in rats (Table 3) [18,30]. Animals were rated on spontaneous movement, symmetry of limb movements, forepaw outstretching, climbing, body proprioception and response to vibrissae touch for a score of 3 to 18 points [20,22,31]. This scale or modifications of it have repeatedly differentiated rats with endovascular SAH from sham-operated controls 6 to 72 hours after SAH [18,20,22,23,31-33]. Other scales developed to measure lateralized deficits after middle cerebral artery occlusion in rats were not shown tested in SAH and sham-operated rats (Table 4) [28,29,34]. The prehensile traction test also was measured in rats with endovascular perforation SAH but without sham-operated animals [28,29,35]. Rats were suspended by their front limbs from a metal rod and the time until falling was measured and treated categorically.

**Table 3:** Behavior Score for Rats with SAH Adapted from Garcia, et al.{Garcia1995}

**Function**		**Score**
Spontaneous activity	Normal	3
	Slightly affected	2
	Severely affected	1
	No movement	0
Symmetry in movement of 4 limbs assessed when rat held suspended by tail	Symmetric	3
	Asymmetric	2
	Hemiplegic	1
Forepaw outstretching assessed by bringing rat to edge of table and making it walk on forelimbs while being held by tail and observing forelimb use	Symmetric forepaws	3
	Mild asymmetry	2
	Marked asymmetry	1
	One forelimb did not move	0
Climbing determined by placing rat on the wall of a wire cage and observing climbing and strength of attachment to wall	Climbed easily, gripped tightly	3
	One side impaired	2
	Failed to climb or tended to circle instead of climbing	1
Body proprioception	Equal on both sides	3
	Reacted slowly to stimulus on 1 side	2
	No response on one side	1
Response to vibrissae touch determined by brushing vibrissae on each side	Symmetric	3
	Asymmetric	2
	No response on 1 side	1
Total		5 to 18

**Table 4:** Rat Neurological Function From Bederson, et al.{Bederson1995}{Bederson1986A}

**Grade**	**Behavior**
Grade 5	Rat held by tail had normal extension of both forelimbs toward the floor
Grade 4	Rat with consistent flexion of forelimb on either side and adduction and internal rotation of shoulder
Grade 3	Rats placed on soft plastic coated paper they could grip with forepaws. With tail held by hand, gentle lateral pressure was applied behind the shoulder until the forelimbs slid several inches. Severely dysfunctional rat with consistently reduced resistance to the paretic side was graded 3
Grade 2	Rats then allowed to move on floor and observed for circling behavior when pulled by tail. Rats circling to paretic side were graded 2
Grade 1	Spontaneous circling when rat allowed to move on floor
Grade 0	No spontaneous motion

Another 16-point scale developed for rats with traumatic brain injury was studied in rats undergoing SAH by endovascular perforation (Table 5)[36,37]. The score combines mobility, neurological reflexes, neurobehavior and beam walking. The neurobehavioral test was seeking behavior. Means for shams were not presented but SAH animals had a score that would probably be significantly different from the normal score of 0 to 1.

**Table 5:** 25 Point Rat Behavior Scale Based on Feldman and colleagues {Feldman1996}

**Characteristic**			**Points**
Mobility	Inability to exit from a circle 50 cm in diameter when placed in center	Within 30 minutes	1
		Within 60 minutes	1
		At > 60 minutes	1
	Hemiplegia (inability to resist forced changes in position)		1
	Inability to walk straight when placed on floor		1
	Inability to move		1
Reflexes	Flexion of hindlimb when raised by tail		1
	Loss of startle reflex		1
	Loss of righting reflex	For 20 min	1
		For 40 min	1
		For 60 min	1
Behavior	Loss of seeking behavior		1
	Prostration		1
Functional tests	Failure in beam walking task	8.5 cm wide	1
		5 cm wide	1
		2.5 cm side	1
	Failure in beam balancing task (1.5 cm wide)	for 20 sec	1
		for 40 sec	1
		for 60 sec	1
	Stability on balance beam (1.5 cm wide)	able to walk, normal gait	0
		able to walk, impaired gait	1
		unable to walk, steady balance on beam	1
		unable to walk, steady balance, all limbs on beam	1
		unable to walk, unsteady balance, unable to place all limbs on beam 1	1
	Effort on beam balance (1.5 cm wide)	unable to stay on the board	1
		unable to try to stay on the board	1
Total			25

Silasi and Colbourne did not detect differences in general activity and forelimb asymmetry in rats undergoing sham or endovascular perforation SAH for up to 21 days after SAH [38]. General activity was decreased to a similar degree after SAH or sham-surgery.

#### Rotarod, Horizontal Ladder and Other Neurological Tests

The rotarod test measures motor function. There are variations in how it is conducted that makes comparison between studies difficult. Thal, et al., placed rats on the device for 10 seconds [29]. Rotation then started and accelerated to 40 revolutions per minute (rpm) within 90 seconds and then remained constant for 30 more seconds. The trial was repeated 5 minutes later and the trial was stopped if the animal fell off or gripped the rungs and spun for 2 revolutions. No sham animals were included. Another method was performed in the double hemorrhage rat model [39]. The rotation was increased from 4 to 40 rpm over 5 minutes for 3 trials per day for 28 days after SAH, sham surgery or saline injection. SAH was associated with marked, persistent deficits for 28 days.

Silasi and Colbourne did not detect differences in tapered beam walking or horizontal ladder function in rats undergoing sham or endovascular perforation SAH for up to 21 days after SAH [38].

#### Beam Balance Test

The beam balance test assesses motor and vestibular function by quantifying the ability to balance on a narrow wooden beam (diameter of 1–2.5 cm) for up to 60 seconds [1,13,14,25,29,40]. Parameters are beam balance time (duration the animal steadily remains on the beam) and beam balance score [13]. Beam balance score is descriptive and examiner-dependent [29].

Rats with single hemorrhage SAH exhibited significantly increased beam balance score 1 day after SAH compared to their function before SAH and to sham-operated and artificial CSF-injected animals [13]. In later studies, the beam balance test was carried out on a wooden beam with a diameter of 1 cm which may increase the sensitivity compared to the 1.5 cm diameter [13].

Most studies using the beam balance were done by one laboratory and although the creation of SAH and behavioral assessments were the same, the results varied, suggesting that the sensitivity is relatively low (Table 1). Deficits usually were detected only in the first 1 to 2 days after SAH [14,24,25]. Variable results may be due to several factors including that the score is subjective and descriptive [13]. The severity of SAH caused by cisternal blood injection also is variable [16]. Finally, the beam balance test is not standardized and there is variability in the diameter, length, shape and composition of the beam which may affect the results [29]. Nevertheless, the results consistently show deficits in the first 24 hours after SAH that tend to resolve after that.

#### Beam Walking Test

The beam walking test is a learned avoidance test. A pre-training session is preceded by a negative reinforcement paradigm in which termination of the adverse stimuli (noise and light) serves as a reinforcement reward. The time taken to traverse the beam and enter a darkened goal box in order to terminate the loud white noise and bright light is measured to assess memory, motivation, attention, somatomotor and locomotor function [13].

Most [1,13,25,40] but not all [14] studies document that rats with SAH created by cisterna magna blood injection take a significantly longer time to traverse the beam compared to before SAH and to sham-operated controls for up to 4 days after SAH. In general, the deficit was maximal 1 day after SAH and then gradually improved. All studies were from one laboratory. Since memory, motivation and attention are involved, this test should be more sensitive to brain injury associated with SAH and this does seem to be the case compared to the other tests described above that assess mainly motor functions.

#### Morris Water Maze

Numerous aspects of learning, memory and neurobehavior can be tested in this apparatus [41]. There are 2 studies employing it after experimental SAH (Table 1)[38,39]. Takata, et al., studied rats undergoing 2 injections of blood or saline into the cisterna magna [39]. Mortality was not reported but would be expected to be high based on prior studies and the massive amount of blood that was injected. Rats were tested for escape latency, swimming speed and swim distance for 16, 60-second trials 29 to 35 days after SAH. The platform was placed in a different quadrant each day and rats were placed randomly in 1 of 4 locations in the maze. If the platform was not found, the rat was placed on the platform for 30 seconds in the first trial or 15 seconds in subsequent trials [42]. The procedure tests learning and short-term memory. SAH was associated with significantly longer escape latency, swim distance and faster swimming speed. Morris water maze testing correlated with neuronal counts in the hippocampus and neocortex.

Silasi and Colbourne compared rats with endovascular perforation SAH to sham-operated animals [38]. They were tested in the Morris water maze from approximately day 21 to 40 after SAH. The procedure was similar to that of Takata, et al., but with 4 trials of 90 seconds per day. SAH rats had longer escape latency and swim distance on days the platform was moved to a new location (every second day). There were Fluoro Jade stained neurons in 4 of 5 SAH rats examined but no other histopathological changes.

### Does Treatment of SAH Affect Neurological Tests?

The only treatments that have reduced mortality in rats undergoing endovascular SAH are hyperbaric oxygen [32] and pifithrin ∀ (Table 1)[20,31]. These studies provide some insight into the complex pathophysiology of brain injury after SAH (Figure 1). These authors hypothesized that hypoxic brain injury at the time of SAH induced apoptosis in large artery endothelial cells by activation of hypoxia-inducible factor 1∀ (HIF1∀). Hyperbaric oxygen decrease expression of HIF1∀ and its target genes, BNIP3 and vascular endothelial growth factor. This was associated with less neuronal injury, improved cerebral blood flow and improved behavior 24 hours after SAH [32]. Apoptosis was inhibited with pifithrin ∀, resulting in less vasospasm and improved blood-brain barrier (BBB) integrity and neurological function [20,31].

Weight loss was assessed in 2 studies. The oxygen free radical scavenger +/- N,N' -propylenedinicotinamide (AVS) did not affect weight loss in rats undergoing single hemorrhage SAH [40]. AVS did improve other endpoints such as vasospasm, balance beam and beam walking, which suggests a role for free radicals in vasospasm and brain injury after SAH and is in keeping with clinical studies showing beneficial effects of AVS [43]. The calpain inhibitor, N-acetyl-leu-leu-methioninal and felbamate prevented body weight reduction for up to 5 days after SAH in the same model [1,25]. Calpains are calcium-activated neutral proteases that may be activated in cerebral blood vessels after SAH, leading to vasospasm and breakdown of the BBB [25]. Thus, preventing their activation may preserve the BBB, as shown by Germano and colleagues [25]. Felbamate has multiple actions that may be neuroprotective including inhibition of voltage-dependent sodium and calcium channels, potentiation of (-amino-butyric acid-mediated chloride currents and reduction of excitatory glutaminergic neurotransmission via N-methyl-D-aspartate receptors [1].

A number of studies demonstrated what would be expected to be beneficial effects on the brain such as neuronal preservation, less vasospasm, less BBB breakdown and/or less brain edema yet found only minimal effects on behavioral testing, suggesting that general neurological scales were not very sensitive to alteration by treatment. The 100 point neurological evaluation or variations of this were different only 1 day after endovascular SAH when treating with hypertonic saline [19] or hypertonic saline plus dextran 70 [29]. Another study comparing infusions of NaCl, mannitol, dextran and hydroxyethylstarch found no differences between groups for up to 7 days after SAH [28]. In all 3 studies, there was less neuronal loss in some of the treated groups at 7 days.

The scale modified from Garcia, et al., differentiated rats 24 hours after endovascular SAH and treatment with caspase inhibitors, hyperbaric oxygen, pifithrin ∀, simvastatin and tetramethylpyrazine [18,20,22,23,32,33]. All treatments improved multiple measures of brain injury. Significant differences persisted for 72 hours among rats treated with pifithrin ∀ compared to dimethylsulfoxide (DMSO) after endovascular SAH [31]. Deficits measured on the scale of Bederson, et al., impairment on the prehensile traction test and rotarod testing, all of which would tend to assess focal motor deficits, were only minimally or not impaired after endovascular SAH or did not differentiate treatment effects [28,29].

The 16-point scale developed for traumatic brain injury [36] detected improved scores in rats undergoing endovascular SAH and treatment with the kinase inhibitor SP600125 compared to DMSO [37]. This scale has advantages of including measures of motor and sensory function as well as beam walking and mobility that may assess higher neurological functions more likely to be impaired after SAH. SP600125, a c-Jun N-terminal kinase inhibitor, decreased neuronal injury and was associated with decreased caspase-3 activation and deoxyribonucleic acid damage, decreased aquaporin 1 upregulation and brain water, reduced matrix metalloproteinase 9 activation and collagen 4 degradation, and preservation of the blood brain barrier (BBB).

Effect of AVS on the beam balance test and BBB function were assessed in rats with single hemorrhage SAH. Continuous infusion of AVS, beginning 5 minutes after SAH, significantly improved BBB integrity, beam balance score and beam balance time 1 and 2 days after SAH and beam traverse time on days 1 to 4 (Table 2)[40]. Similar results were reported by Imperatore, et al. [14]. Other pharmacologic treatments that significantly improved beam balance scores for 1 to 3 days after single hemorrhage SAH were the calpain inhibitor, N-acetyl-leu-leu-methioninal [25] and felbamate [1]. Other investigators questioned the sensitivity of the beam balance test [29]. These investigators used endovascular SAH instead of cisterna magna single injection and the rats were randomly assigned to groups of control (intravenous 0.9% NaCl), moderately neuroprotective therapy (intravenous 7.5% NaCl) and highly effective neuroprotection (intravenous 7.5% NaCl + 6% dextran 70). The beam balance test employed differed from prior studies and comprised 2 wood rods (1.5 and 2.5 cm diameter) positioned horizontally 40 cm above a foam pad. Rats were placed on the center of each beam and the time they spent on the rod was recorded up to a maximum of 120 seconds. There were no differences between groups. Although no sham operated animals were included, rats were tested before SAH and there were only minimal deficits in the first 2 days after SAH.

The beam walking test differentiated placebo from treatment effects of AVS [24] and a calpain inhibitor [25] for 1 to 4 days post-SAH and felbamate treatment for 2 days [1]. Imperatore and colleagues did not find significant effects of AVS, however [14].

Some differences in results may be due to the model used. While the injection model may be more applicable to investigation of the direct effects of hemorrhage and delayed pathophysiological events like cerebral vasospasm, and hence more enduring behavioral deficits, the perforation model resembles human pathophysiology of aneurysmal rupture. The high mortality rate parallels the human situation and the model has been used to investigate early pathophysiological changes immediately after SAH. The subtle neurological alterations in the perforation model correlate with reports that neuronal death in the perforation model was seen in 11% of rats compared to 28% of rats undergoing cisterna magna injection SAH [16].

### Correlation with Vasospasm

The pancaspase inhibitor z-VAD-FMK decreased TUNEL and caspase 3 in endothelial cells, decreased caspase 3 activation, reduced BBB permeability and brain edema, improved neurological outcome and decreased vasospasm after endovascular SAH [18]. Cahill and colleagues also reported better neurological scores, less brain edema and BBB breakdown, less vasospasm and less basilar artery apoptosis after treatment with pifithrin ∀ [20]. On the other hand, Takata, et al., used a double hemorrhage rat SAH model and found deficits in rotarod, vertical screen and balance beam, Morris water maze, as well as chronically decreased cerebral blood flow, neuronal loss in the hippocampus, and microvascular filling defects despite minimal proximal large artery vasospasm [39].

Therefore, there is some evidence that brain injury can occur after SAH without vasospasm. This is not surprising, nor are findings of a lack of correlation between vasospasm and any other endpoint. There are multiple pathways to poor outcome after SAH, only one of which is vasospasm (Figure 1). The relative importance of each will vary depending on the model and treatment may affect only one pathway [44]. Furthermore, the relationship may not be linear so simple statistical tests may not detect correlations.

### What Other Tests Could be Used?

A limitation of most of the neurological tests used is they were developed to detect deficits produced by focal ischemia, usually of the middle cerebral artery territory. While this can occur after SAH, it is not common and more patients have deficits in neurobehavioral function, such as memory, visuospatial/construction ability and executive function than focal neurological deficits [45,46]. Focal deficits are rare in animal models of SAH. Tests that would be more sensitive to the effects of SAH in animals could be selected based on regions of the brain known to be damaged after experimental SAH and/or based on deficits that occur more commonly after SAH in humans. Detailed evaluations of the regions of brain damaged after SAH in humans and experimental animals are sparse, however.

After SAH created by cisterna magna injection in rats, opening of the blood-brain barrier in cerebral cortex, brainstem and cerebellum was noted [47]. Vasospasm is most marked in the basilar artery and neuronal death may occur in the hippocampus and striatum [15,16]. Cerebral blood flow reductions 15 minutes after SAH were diffuse but most marked in the brainstem and cerebellum [48]. Neuronal loss was reported in neocortex and hippocampal CA1 [39].

After SAH created by endovascular perforation, there also was diffuse opening of the BBB. Neuron apoptosis or increased messenger ribonucleic acid for proteins involved in apoptosis was reported in basal (orbital, cingulate, prefrontal) cortex [38,49], hippocampus [50], CA1 [16] and Nissl staining showed neuronal injury in hippocampus (CA1 to CA3), motor cortex, caudoputamen and cerebellum bilaterally [28,29]. The changes are greater on the side of endovascular perforation. Vasospasm was centered in the ipsilateral anterior circulation and reduced cerebral blood flow 15 minutes after SAH was bilateral and diffuse but most marked in the anterior circulation [48]. Findings were similar in the chiasmatic cistern injection model except that vasospasm is bilateral and severest in the anterior circulation, BBB changes are diffuse and neuronal injury was reported in prefrontal and cingulate cortex, thalamus, striatum and hypothalamus [16,48]. In some studies, minimal neuronal injury occurs or it is only observed in some animals so it is likely that sensitive tests of neurobehavior would be required [38,49].

Neurobehavior tests used in rodents have been used to assess attention, learning, memory and cognition (Table 6) [51-53]. The 5-choice serial reaction task and reaction time procedures measure attention, which is frequently impaired after SAH in humans [54]. Active avoidance conditioning paradigms, such as fear conditioning, may assess basal frontal lobe function, which may be damaged after SAH [55].

**Table 6:** Other Neurobehavior Tests for Potential Use in SAH Studies

**Tests**	**Species**	**Neurobehavior Assessed**	**Measures**	**Methods**
Five-choice serial reaction task	Rat	Attention	Steady-state procedure in which the effects of various neural and behavioral manipulations are examined on a baseline of stable attention performances	Rat is required to detect brief flashes of light occurring in one of the 5 holes in order to earn food pellets

Reaction time procedure	Rat	Attention	rat's response to visual stimuli while its head is in a fixed location- time it takes for the rat to withdraw its head from the central location and thus cease to break the vertical photocell beam	rat is trained to hold its head in a central location by interrupting the photocell beam there. Brief visual stimuli are presented to either side of the rats head

Active avoidance conditioning paradigm (eg. Fear conditioning)	Rat	Learning/memory to avoid noxious stimulus	Escape or avoidance latencies	Rat is trained to avoid noxious stimulus by withdrawing itself from the source of the stimuli (eg. Foot shock)

Nonmatching to sample (NMTS)/matching to sample tests (MTS, can be either spatial or non-spatial)	Rat	Working memory test (trial-unique)	Latency to make the choice/error in choice (either to pick the same [MTS] or alternative [NMTS] stimulus)	Rat is pre-trained either to choose (on test trial) the same or alternative stimulus which is shown on sample trial

Delayed NMTS/MTS tests	Rat	Short-term memory	Latency to make the choice/error in choice (either to pick the same [MTS] or alternative [NMTS] stimulus)	Same as NMTS/MTS tests except they introduce various inter-trial intervals

Radial-arm maze	Rat	Spatial working memory	Errors in first 10 choices, total errors/session	Food-deprived rats trained to learn to avoid choosing arms (8 arms with food baited in one arm) they already visited (where there are no food pellets) as they learn the spatial location of each arm and remember the locations they had visited

Open Field	Rat	Exploratory and locomotor activity	Locomotion (number of square crossings), rearing, grooming, stereotypical behaviors (licking, biting, head weaving)	Video camera positioned above open field to consistently record behavior of rodents in the open field apparatus

Perceptual attentional set shifting task	Rat	Complex attention	Reversal/set shift task where rat required to discriminate which of 2 bowls has food based on variations in odor and texture of the medium the food is in	Number of trials and errors to learn location of food

Morris water maze	Rat	Spatial learning and memory	Escape latency, swimming distance, time spent in each quadrants, annulus crossing numbers	Animals are allowed freely swim to find a platform in swimming pool, guided by extramaze cues that surround the pool

Eyeblink classical conditioning	Rabbit	Associated learning	Number of paired trials required to reach the learning criterion (eg. 8 conditioned responses in 9 consecutive trials)	One eye held open. Conditioned stimulus such as a sound presented after unconditioned stimulus such as corneal airpuff. Paired trial present throughout the training. Minitorque potentiometer measures nictitating membrane/eyeblink response.

Open field	Rabbit	Behavioral reactivity	Movement activity (eg. Jump, rearing, locomotor, grooming), social behavior, aggressive behavior (strong blows with the hindpaws), emotional tension (number boluses), passive-defensive behavior (freezing time)	Video camera positioned above open field records behavior of animal in the open field apparatus during a specific time period

Discriminative avoidance/approach task	Rabbit	Cognition	Number of training sessions required for animals to attain the criterion	Rabbits learn to prevent a foot-shock by stepping in a large activity wheel in response to a shock-predictive tone and they ignore different tone which does not predict the shock

Delayed-non-matching-to-position (DNMP)	Dog	Visuospatial learning/memory and working memory	Response-choice latency on the test trial	Animals are allowed to displace the red block and retrieve the food reward beneath the block on the sample trial. Animals are permitted access to the food reward by displacing the block over the non-match position on the test trial (inter-trial interval varies for working memory)

Open field	Dog	Exploratory and locomotor activity	Exploratory behavior, locomotion, inactivity, sniffing, urinating, jumping, rearing, vocalization	Video camera positioned above open field records behavior of animal in the open field apparatus during a specific time period

Object discrimination task	Dog	Working memory	Performance accuracy	Two wooden blocks that were identical except for color present as stimuli (eg black and white). Dogs are pre-trained to approach one of the two blocks to obtain food reward. Testing is repeated after SAH

Reversal task (usually followed by object or size discrimination task)	Dog	Executive function (inhibitory control, performance monitoring-eg reversal learning)	Total number of errors	Two identical wooden blocks in color and material, different only in size present as stimuli. Dogs learn the size preference for the food reward, followed by reversal learning in which the reward contingencies of positive and negative block are reversed

Several studies suggest memory is impaired after SAH [45,46,56]. The specific aspects of memory affected vary but many can be assessed in rodents [53]. Many paradigms are available for the Morris water maze [57]. Nonmatching (NMTS) and matching-to-sample (MTS) tests that can be spatial or non-spatial have been described and the time between trials can be delayed to test short-term memory [58]. Open field behavior also is used to assess neurobehavior. Frontal lobe function can be measured by the perceptual attentional set shifting task [59,60].

It should be recognized that tests for specific neurobehaviors in humans are available and that just as classic neurological tests can localize various motor and sensory functions, sophisticated neurobehavior tests may localize to discrete brain regions. For example, ventromedial frontal cortex damage may be detected specifically by tests like the Iowa gambling task [61]. Marked abnormalities can occur when the minimental status examination is normal [62]. Tests that specifically test discrete areas of rodent cortex are less well documented. Some functions in humans and rodents, like anxiety and startle responses, may be mediated by diffuse neural networks. Another problem is that there is variability in the areas of the brain damaged by SAH so multiple tests might be needed but this is not easy to do in humans or experimental animals [46]. At this point it is difficult to make firm recommendations on what tests should be used in rodents with SAH. Preliminary choices might be those assessing attention, short-term working memory and basal frontal lobe function that probably involves olfaction in rodents and might be tested by perceptual attentional set shifting task.

## Mice

### Models

The 2 most commonly used models are the same as used in rats; single injection of blood into the cisterna magna or endovascular perforation [63-65].

### Endpoints Used

Motor and sensory activity were assessed in the endovascular perforation model on a scale combined from 2 prior scales and comprised motor (spontaneous activity, symmetry of limb movements, climbing, balance and coordination for 0–12 points) and sensory (proprioception, vibrissae, visual, olfactory and tactile responses for 5–15 points, Tables 7 and 8)[30,63,66]. Mortality was not reported. There was significant weight loss 3 days after SAH compared to sham-operated animals. Neurological function was significantly impaired compared to sham-operated mice. Several variations of this scale were assessed 72 hours after endovascular SAH [67,68]. It differentiated sham from SAH animals and also differentiated animals treated with simvastatin or vehicle [67] but not between wild-type and transgenic mice overexpressing human extracellular superoxide dismutase [68].

**Table 7:** A Mouse Motor and Sensory Scale {Parra2002}.

**Function**		**0**	**1**	**2**	**3**
Motor					
	Activity (5 minutes open field)	No movement	Moves, no walls approached	1-2 walls approached	3-4 walls approached
	Limb symmetry (suspended by tail)	Left forelimb, no movement	Minimal movement	Abnormal forelimb walk	Symmetrical extension
	Climbing (on inverted metal mesh)	Fails to hold	Hold < 4 seconds	Holds, no displacement	Displaces across mesh
	Balance	Falls < 2 seconds	Falls > 2 seconds	Holds, no displacement	Displaces across rod
Sensory	Proprioception (cotton tip to both sides of neck)		No reaction	Asymmetrical head turning	Symmetric head turning
	Vibrissae (cotton tip to vibrissae)		No reaction	Asymmetrical head turning	Symmetric head turning
	Visual (tip toward each eye)		No reaction	Unilateral blink	Bilateral blink
	Olfactory (lemon juice on tip)		No sniffing	Brief sniff	Sniff > 2 seconds
	Tactile (needle stick to palm)		No reaction	Delayed withdrawal	Immediate withdrawal

**Table 8:** Selected Studies of SAH in Mice Examining Mortality and Neurological Endpoints

**Author**	**Model**	**Mortality**	**Behavior tests**	**Controls**	**Experimental findings**	**Disconnect between vasospasm and outcomes**
Parra2002	Endovascular perforation	none reported	Neurobehavior score of 5-27 72 hours after SAH, combined from 2 prior scales {Garcia1995}{Crawley2000}, comprised of motor (0-12) spontaneous activity, symmetry of limb movements, climbing, balance and coordination and sensory (5-15) which was proprioception, vibrissae, visual, olfactory and tactile responses.	SAH or sham-operated controls	72 hours after SAH, body weight reduced in SAH group, neurological function worse, correlated with vasospasm and SAH grading.	They correlated
McGirt2002	Endovascular perforation	6/34 (18%) simvastatin versus 4/36 (11%) vehicle which would not be significant (Fisher's exact test) by my calculations	Neurobehavior score of 5-27 72 hours after SAH, combined from 2 prior scales {Garcia1995}{Crawley2000}, comprised of motor (0-12) spontaneous activity, symmetry of limb movements, climbing, balance and coordination and sensory (5-15) which was proprioception, vibrissae, visual, olfactory and tactile responses.	Simvastatin versus vehicle	More vasospasm and behavior deficit with SAH compared to shams and simvastatin prevented vasospasm and behavior deficits.	They correlated
McGirt2002A	Endovascular perforation	9% mortality, no statement about if it was different between groups	Neurobehavior score of 9-39 72 hours after SAH, combined from 2 prior scales {Garcia1995}{Crawley2000}, comprised of motor (0-12) spontaneous activity, symmetry of limb movements, climbing, balance and coordination and sensory (5-15) which was proprioception, vibrissae, visual, olfactory and tactile responses and reflexes (4-12) righting, postural, ear and eye	SAH in wild-type, human extracellular superoxide dismutase transgenics and sham-operated controls	More vasospasm and behavior deficit with transgenics and wild types compared to shams of both strains but no difference between transgenic and wild type, less nitrotyrosine staining in the transgenics, body weight did not differ between transgenic and wild type but unclear if this was at baseline or after SAH.	They correlated
Lin2003	Single 60 μl cisterna magna injection over 1 minute, no ICP monitoring	3%	None	SAH, saline-injected and sham-operated controls	Vasospasm could be produced, no behavior assessment	Not assessed
Gao2006	Endovascular perforation	18% of apoe3 versus 38% of apoe4 mice	Rotarod, neurological severity score consisting of motor (spontaneous activity, symmetry of limb movements, climbing and balance and coordination), sensory (body proprioception and tactile and vibrissa responses)	SAH in apoe3, apoe4, apoe4 mimetic peptide treated and sham-operated controls	SAH groups had significantly worse behavior than sham-operated controls. Among apoe animals, there was better rotarod performance and less vasospasm with apoe3 mice compared to apoe4. Apoe4 peptide mimetic reduced mortality, improved neurological score, rotarod latency and vasospasm	They correlated
Wang2006	Endovascular perforation	None reported	Rotarod, neurological severity score consisting of motor (spontaneous activity, symmetry of limb movements, climbing and balance and coordination), sensory (body proprioception and tactile and vibrissa responses)	SAH and sham-operated controls but no results of sham-operation reported	Levetiracetam improved rotarod, neurological score and vasospasm	They correlated
Mesis2006	Endovascular perforation	None reported	Rotarod, neurological severity score consisting of motor (spontaneous activity, symmetry of limb movements, climbing and balance and coordination), sensory (body proprioception and tactile and vibrissa responses)	None	Carboxyamidotriazole worsened behavioral outcome and decreased vasospasm whereas nimodipine and apo E mimetic peptide improved neurological scores, rotarod latency and decreased vasospasm.	Yes, carboxyamidotriazole worsened function and decreased vasospasm which could be due to drug toxicity, only low dose nimodipine decreased vasospasm and improved outcome whereas high dose did not and the authors suggest this proves vasospasm does not cause all deficits after SAH which is true but examination of bar graphs shows a 2 μm difference (about 2%) between the nimodipine doses

Mortality was reported in several mouse SAH models. Cisterna magna injection of autologous blood, 60:l, was associated with 4% mortality with no mortality mentioned for saline-injected animals [65]. In an endovascular perforation model, mortality (27 to 29%) did not differ between wild-type and human CuZn superoxide dismutase overexpressing transgenic mice [64]. Body weight changes have not been studied.

Mesis and colleagues studied rotarod performance and a neurological score also used by McGirt, et al., with minor differences in mice with perforation-induced SAH [69]. The neurological score was a subset of tests from a 48-point scale developed from prior scales for rats and mice [30,70-73]. Mortality was not reported. Animals were tested before SAH and there appeared to be significant deficits in rotarod performance and neurological scores for 3 days after SAH.

### Does Treatment of SAH Affect Neurological Tests and Correlate with Vasospasm?

Treatment of mice with perforation-induced SAH with high-dose carboxyamidotriazole, a voltage and nonvoltage-dependent calcium channel inhibitor, worsened rotarod performance, decreased vasospasm and was associated with a trend to worse neurological score whereas a low dose did not affect rotarod or neurological function or vasospasm compared to vehicle-treated animals [69]. Another series of mice were treated with 2 doses of nimodipine which improved neurological scores, rotarod latency and decreased vasospasm. There was about a 2% (2:m) mean diameter difference in vasospasm between the nimodipine doses that resulted in the effect on vasospasm being insignificant in the low-dose group. An apoE mimetic peptide, acetyl-AS-Aib-LRKL-Aib-KRLL-amide, administered alone or with nimodipine, also improved all 3 endpoints [74]. Several interpretations are possible. One is that carboxyamidotriazole has toxic effects at high doses that worsen behavior. The beneficial effects of nimodipine may be separate or in addition to decreasing vasospasm [75]. ApoE mimetic peptides were neuroprotective in other brain injuries and may decrease vasospasm via antiinflammatory mechanisms [74]. In support of this, the same behavior endpoints were assessed in mice that express only human *APOE3 *or *APOE4 *[74]. After SAH created by endovascular perforation, mice with *APOE3 *had better rotarod performance and less vasospasm compared to *APOE4 *mice. An ApoE4 peptide mimetic administered to wild-type mice after SAH reduced mortality and improved neurological score, rotarod latency and vasospasm.

Another report from the same laboratory found that levetiracetam had the same pattern of effects in that it improved all 3 measures in high doses in SAH mice [76]. Levetiracetam may be neuroprotective and antivasospastic by virtue of its ability to inhibit voltage-dependent calcium, (-aminobutyric acid and glycine currents [76].

### What Other Tests Could be Used?

Few studies have examined areas of brain injured after SAH in mice. Learning, memory and neurobehavior assessment in mice, while not identical, is similar to in rats and has not been assessed yet after murine SAH. Recommendations would probably be similar to those for rats.

## Rabbits

### Models

This is limited to cisterna magna injections of blood once or twice [77,78]. SAH has been combined with ligation of the carotid arteries in an attempt to induce cerebral ischemia from vasospasm [79].

### Endpoints Used

Endo, et al., ligated both common carotid arteries and 2 weeks later induced SAH by injecting blood into the cisterna magna of rabbits (Table 9)[79]. Neurological deficits were categorized as normal, minimal or suspicious neurological deficit, mild deficit without abnormal movement or severe neurological deficit with abnormal movement. 5 of 13 animals had mild dysfunction after carotid ligation and this was more severe after SAH and more severe than in animals with SAH alone. Subtle transient decrements in neurological function were detected in rabbits with SAH treated with intravenous anticardiolipin antibodies compared to those with SAH alone [80]. Whether nimodipine and ecdysterone improved neurological function on this scale in another study was difficult to discern from the paper [81]. A more complicated model added, in addition to bilateral carotid occlusion and SAH, oxyhemoglobin cisternal injection 2 days after SAH [82]. No correlation between vasospasm and neurological score was detected although the degree of vasospasm was similar for grades 2 and 3 and worse for grade 4.

**Table 9:** Selected Studies of SAH in Rabbits Examining Mortality and Neurological Endpoints

**Author**	**Model**	**Mortality**	**Behavior tests**	**Controls**	**Experimental findings**	**Disconnect between vasospasm and outcomes**
Endo1988A	Single cisterna magna injection plus bilateral carotid occlusion	None reported after SAH, some animals died after carotid occlusion	4 point neurological grading scale consisting of 1. No neurologic deficit (normal), 2. Minimum or suspicious neurologic deficit, 3. Mild neurologic deficit without abnormal movement, 4. Severe neurologic deficit with abnormal movement	SAH or saline-injected controls	Worse behavior and production of vasospasm in SAH compared to saline-injected controls	They correlated
Otsuji1994A	Bilateral carotid occlusion, then 2 weeks later SAH followed 2 days later by cisternal injection of oxyhemoglobin	8/23 died (32%) after the second injection	4 point neurological grading scale consisting of 1. No neurologic deficit (normal), 2. Minimum or suspicious neurologic deficit, 3. Mild neurologic deficit without abnormal movement, 4. Severe neurologic deficit with abnormal movement	None	Neurological deficits in some animals	No correlation reported between neurological grade and vasospasm, there was a better correlation between CBF and neurological grade. Grade 2 and 3 had about 25% vasospasm and grade 4 had 40% so at least the most markedly worse neurological grade had more vasospasm
Nomura1998	Bilateral carotid occlusion, then 5 weeks later SAH by single cisterna magna injection	0/9 SAH, 3/8 SAH + immunization with subcutaneous cardiolipin antigen, 5/12 SAH + intravenous cardiolipin antigen, 0/8 SAH plus intravenous cardiolipin antigen + dexamethasone + cyclosporin A	4 point neurological grading scale consisting of 1. No neurologic deficit (normal), 2. Minimum or suspicious neurologic deficit, 3. Mild neurologic deficit without abnormal movement, 4. Severe neurologic deficit with abnormal movement	None	Neurologic deficits and vasospasm worse with SAH plus intravenous cardiolipin antigen compared to SAH alone whereas cyclosporin + dexamethasone reversed this to the SAH alone level	They correlated
Buemi2000	Single cisterna magna injection, no ICP monitoring	0% with control or SAH + erythropoeitin, 43% SAH + vehicle	Open field locomotor activitiy	Probably normal rabbits	Erythropoeitin improved locomotor activity, one comment that that there was no corrugation of the internal elastic lamina in animals treated with erythropoeitin	Not assessed
Grasso2002	Single cisterna magna injection, no ICP monitoring	None reported	Daily 4 point neurological assessment of normal (1), minimal or suspected deficit (2), mild deficit (3) or severe deficit with abnormal movements (4)	SAH or control, unoperated animals	Erythropoeitin improved neurological status, decreased necrotic cortical neurons and vasospasm	They correlated
Zhou2007A	1 or 2 injections into cisterna magna, 1.5 ml blood once or twice over 1 minute, no ICP monitoring	0% single, 6% double hemorrhage	Vasospasm, mortality, clinical assessment by the 6 point scale {Zhou2005}	SAH or control, unoperated animals	Only significant behavior difference was poor appetite in double hemorrhage group, vasospasm in both groups, a little less with single hemorrhage	They correlated
Laslo2008	Single cisterna magna injection, no ICP monitoring	10/25 (40%) SAH died, no sham-operated controls died	Vasospasm and neurological scale of posture, gait, and righting reflexes (each given a score: 0 normal, 1 mild, 2 moderate and 3 severely impaired. Front and back reflexes were also scored 0 normal, 1 brisk, 2 spreading and 3 clonus	SAH or saline-injected controls	Neurological function worse with SAH and with more severe vasospasm	They correlated
Tang2008	Right common carotid artery ligation + single cisterna magna blood injection	None reported	4 point neurological grading scale consisting of 1. No neurologic deficit (normal), 2. Minimum or suspicious neurologic deficit, 3. Mild neurologic deficit without abnormal movement, 4. Severe neurologic deficit with abnormal movement	None	Neurological function and vasospasm decreased by ecdysterone	They correlated

Mortality and open-field locomotor activity were assessed after single SAH in rabbits and compared in animals given intravenous saline or erythropoeitin [77]. No animals underwent sham surgery. Mortality was reduced significantly after SAH when erythropoeitin was administered. Open-field locomotor activity was assessed by number or rearings and a measure of the amount of movement about an open field apparatus and was improved also with erythropoeitin.

The 6-point scale developed for dogs was applied to rabbits with SAH created by one or 2 injections of blood into the cisterna magna [78]. The only significant difference was the appetite score was significantly higher 3 days after double SAH compared to single hemorrhage. There were no saline-injected controls.

Mortality rates and weight changes are not well-described in rabbit models of SAH. One group reported 40% mortality after single cisternal injection SAH in rabbits compared to 0% after saline injection [83] and another had 0% after single and 6% after double injection SAH [78].

Another neurological scale developed to assess myelopathy in rabbits was applied to rabbits undergoing SAH. The scores were significantly worse in animals with severe delayed vasospasm compared to those with mild vasospasm or sham surgery [83]. The neurological functions evaluated were posture, gait, and righting reflexes (each given a score of 0 for normal, 1 for mild impairment, 2 for moderate impairment and 3 for severe impairment. Front and hindlimb reflexes were scored 0 for normal, 1 for brisk, 2 for spreading and 3 for clonus.

### Does Treatment of SAH Affect Neurological Tests and Correlate with Vasospasm?

There were no reports of a lack of relationship between the severity of vasospasm and neurological function in rabbit models of SAH.

### What Other Tests Could be Used?

Some other tests used in rabbits include eyeblink conditioning and the discriminative avoidance/approach task [84,85]. Open field activity was assessed in one study already [77,86]. The disadvantages of using a rabbit model of SAH would be that there are fewer behavior tests, in addition to limited availability of specific molecular biological reagents for assessing other endpoints.

## Dogs

### Models

SAH has been produced most commonly by one or 2 injections of blood into the cisterna magna.

### Endpoints Used

Several studies assessed neurological function within hours of SAH or used only broad qualitative assessments (Table 10) [87-89]. A single injection model in dogs used a 6 point neurological function scale. There were no saline-injected controls. Some nonsteroidal antiinflammatory drugs decreased vasospasm 24 hours after SAH and improved scores on this scale [89,90]. Other similar scales including a dog coma score did not detect deficits after SAH in dogs [91,92].

**Table 10:** Selected Studies of SAH in Dogs Examining Mortality and Neurological Endpoints

**Author**	**Model**	**Mortality**	**Neurological and Neurobehavioral Tests**	**Controls**	**Experimental findings**	**Disconnect between vasospasm and outcomes**
White1979	Single cisterna magna injection of 2-4 ml without ICP monitoring	None reported	Whether the animal ate, neurological deficits and change in demeanor	None	Saline treatment for vasospasm gave 46% behavior abnormalities, versus 11% for sudoxicam treatment	Some relation between behavior and vasospasm but authors reported that there was no absolute correlation.
White1979A	Single cisterna magna injection of 2-4 ml without ICP monitoring	None reported	Whether the animal ate, stood up, central nervous system depression, paresis and ataxia	Some animals assessed after angiography only	SAH produced more behavior change than angiography alone	Phenoxybenzamine treatment did not decrease vasospasm, no correlation between vasospasm and behavior
Chyatte1983	Double hemorrhage model, ICP not measured	11/36 (31%) eliminated from study, including 5 deaths as result of initial anesthesia, 3 after injection of blood and 3 angiographic complications	Brief qualitative mention of meningeal signs and neurological deficits	None	9/9 with SAH had meningeal signs and this was significantly less with drug treatment (2/8 treated with ibuprofen and 2/8 treated with methylprednisolone). Neurological deficits in all groups but seemed to improve faster with drug treatment, no statistical analysis	Vasospasm correlated with meningeal signs and behavior
Varsos1983	Double hemorrhage model, ICP not measured	None reported	Brief mention that no dogs developed hemiparsis, some were drowsy and had staggering gait on day 5	None	Aminophylline, nifedipine and papaverine at single tested doses did not reverse vasospasm	Not assessable
White1983	Single cisterna magna injection of 2-4 ml without ICP monitoring	None reported	6 level scale of no neurological deficit (0), lethargic/decreased motor activity (1), paresis/staggering gait (2), failure to eat (3), failure to walk (4), failure to stand (5)	None	Some benefit of nonsteroidal antiinflammatory drugs for early vasospasm 1 and 24 hours after SAH	No relationship between behavioral abnormalities and vasospasm 24 hours after blood injection altthough the average vasoconstriction increased with increased behavior abnormality (r = 0.44, p < 0.01)
Alexander1985	Double hemorrhage model, ICP not measured		The neurological findings were graded from 0 to 5, based on meningismus, ataxia, paresis, and cranial nerve deficits. No significant differences in neurological grade were found on any day between the two groups.	None	No effect of lavage on vasospasm or behavior	No correlation between neurological findings and vasospasm
Diringer1991	4-5 ml blood or saline injected 2 to 3 times into cisterna magna, ICP not measured	None reported	Brief description of behavior	SAH, saline injected and controls	General observations that SAH was associated with animals being less alert and having decreased appetite	Not assessable
Kaoutzanis1993	Single and double hemorrhage models, ICP not measured	None reported	11-point coma scale assessing motor response, eye movements and food intake	None	All dogs had normal consciousness regardless of degree of vasospasm	Not assessable
Tibbs2000	Double hemorrhage model, ICP not measured	1 of 22 (5%)	3 categories - 1. dog active with normal appetite and no focal neurological deficits, 2. not active, poor appetite or somnolent or 3. focal neurological changes such as ataxia or hemiparesis	None	PD-98059, a mitogen-activated protein kinase inhibitor, decreased vasospasm and improved behavior compared to dimethyl sulfoxide and controls. U-0126 was not effective against vasospasm but did improve behavior.	U-0126 did not prevent vasospasm but improved behavior
Zhou2004	Double hemorrhage model, ICP not measured	None reported	6 point neurological grading based on appetite, activity and neurological deficits	None	Caspase inhibitors Ac-DEVD-CHO and Z-VAD-FMK prevented vasospasm and improved appetite and activity. SAH had almost no effect on neurological function	By day 7 behaviour was similar among groups despite decreased vasospasm with caspase inhibitors
Yamaguchi2004B	Double hemorrhage model, ICP not measured	None reported	6 point neurological grading scale based on appetite, activity and neurological deficits	None	Ras farnsyl transferase (FTAse) inhibitor (FTI-277) and FTAse inhibitor 1 prevented vasospasm and improved activity and appetite but had no effect on neurological function which was normal in most animals	They correlated
Yamaguchi2004A	Double hemorrhage model, ICP not measured	None reported	6 point neurological grading scale based on appetite, activity and neurological deficits	None	Adenovirus expressing superoxide dismutase or lac Z did not prevent vasospasm and was associated with no effect on neurological and neurobehavioral ouotcome expect for worse appetite score with superoxide dismutase treatment 1 day after SAH	They correlated
Yatsushige2005	Double hemorrhage model, ICP not measured	None reported	6 point neurological grading scale based on appetite, activity and neurological deficits	None	C-jun N-terminal kinase (JNK) inhibitor SP600125 improved behavior and reduced vasospasm in dose-dependent manner	They correlated
Zhou2005	Double hemorrhage model, ICP not measured	None reported	6 point neurological grading scale based on appetite, activity and neurological deficits	None	Intracisternal p53 inhibitor, pifithrin alpha, improved appetite and activity several days after SAH, no effect on neurological deficits (most dogs were normal), less apoptosis and decrease in vasospasm	They correlated

The most widely used scale assessed appetite, activity and neurologic deficits in the double injection model [93-98]. Appetite was graded as finished meal = 2, left meal unfinished = 1, scarcely ate = 0. Activity was graded as active, barking or standing = 2, lying down, will stand and walk with some stimulation = 1, almost always lying down = 0. Neurological deficits were graded as no deficit = 2, unable to walk because of ataxia or paresis = 1, impossible to walk or stand because of ataxia or paresis = 0. We could not find reports of whether the scale differentiates saline-injected controls from SAH. Mortality is seldom reported but is low in this model and not significantly different between SAH and saline-injected controls (R.L. Macdonald, personal observation). Weight has not been used as an endpoint. The scale detected significant improvement in appetite and activity after treatment with mitogen associated protein kinase inhibitor [93]. Caspase inhibitors Ac-DEVD-CHO and Z-VAD-FMK improved appetite within 3 days of SAH and produced variable improvements in activity [99]. These results confirm rat studies suggesting inhibition of endothelial cell apoptosis decreases vasospasm.

Other treatments that were associated with similar improvements were inhibitors of Ras FTase [100], JNK [101] and p53 [102], again supporting a role for apoptosis and signal transduction in the artery wall mediating vasospasm. The neurological deficit portion of the scale was almost never affected.

### Does Treatment of SAH Affect Neurological Tests and Correlate with Vasospasm?

No relation between neurological deficits measured on a 6-point scale and vasospasm was claimed although there was a general increase in vasospasm with worsening neurological score [89,90]. The scale also is altered by pain that would be reduced by the drugs independent of any effects on vasospasm or brain injury.

U-0126 improved behavior but vasospasm was not decreased [93]. U-0126 is a mitogen-activated protein kinase kinase inhibitor that decreases arterial contractions to endothelin and erythrocyte hemolysate. The authors hypothesized that neuroprotective effects might account for the improved behavior.

### What Other Tests Could be Used?

In the single and double-hemorrhage models, vasospasm is most severe in the basilar artery and less marked in the anterior circulation [103]. One study noted caspase-3 and glial fibrillary acidic protein in astrocytes and neuronal injury were most marked in the hippocampus, second in the cortex and least in the brainstem in the double-hemorrhage dog model [104]. Tests of learning and memory might therefore be worth assessing in this model. There are numerous sophisticated neurobehavior tests available for dogs, including open field behavior, object discrimination tasks often with reversal tasks and various permutations of immediate and delayed nonmatching-to-sample tests [52,105]. These assess attention, executive function, complex learning and spatial learning and are sensitive to aging and interventions [106-108].

## Summary and Future Directions

High mortality is a characteristic of SAH in humans and it has been assessed after SAH in rats and mice in several models and is higher after SAH than sham-surgery (Table 11). This endpoint is not well-described in rabbits and dogs. Mortality is low in the dog double hemorrhage model because significant vasospasm can be produced without having to produce an injury severe enough to be fatal. Several treatments have reduced mortality in rodent SAH models and this endpoint should be reported and probably included in outcome analysis.

**Table 11:** Summary of Neurological and Behavior Tests After Experimental SAH

**Test**	**Advantages**	**Disadvantages**	**Differentiates Sham from SAH**	**Detects Treatment Effects**
Mortality	Quantitative, easy to measure	Treatments in humans save some patients whereas animals die and these may be the most likely to have neurological and neurobehavior deficits	Significantly different for endovascular SAH in mice and rats, injection model in rabbits, not in dogs	Decreased mortality after endovascular SAH with some treatments in rats, not reported in other species
Body weight	Quantitative, easy to measure	May not be very sensitive to effects of SAH	Transiently decreased after SAH in rats and endovascular SAH in mice, not described in dogs	Less weight loss after endovascular SAH with some treatments in rats, not described in dogs
Neurological Function	May detect delayed cerebral ischemia that is an important clinical event, widely used in other types of brain injuries	Often qualitative, focal motor, sensory and reflex alterations are not common after clinical or experimental SAH	Several scales detect differences after endovascular SAH in rats, mice and rabbits {Katz1995}{Garcia1995}{Parra2002}, rotarod may detect differences after double hemorrhage SAH in rats and sinble hemorrhage SAH in rats may produce differences in beam balance and beam walking. Not assessed in dogs	Several scales detect treatment effects after endovascular SAH in rats and mice {Garcia1995}{Feldman1996}{Parra2002}. Beam balance and beam walking may improve with treatments after single SAH in rats within first days. Appetite and activity may be improved by treatments after double SAH in dogs, neurological deficits almost never observed
Neurobehavior tests	Executive function, memory, learning, attention are commonly affected after clinical SAH, tests can be selected to assess very specific neurobehaviors or discrete brain regions of interest	More complicated and expensive to use, since they have not been used much in SAH the sensitivity and specificity are unknown, may require lack of impairment in neurological function to perform	Impaired learning and memory after double injection or endovascular SAH in rats, not assessed in any other models	Not assessed

Body weight decreases after SAH in rats and mice but has not been assessed in other animal models. These decreases can also be mitigated by treatment in some cases. Neurological scales testing motor, sensory and reflex functions have been mainly used in rats, mice and dogs and can detect effects of SAH although the differences are often small and transient. Rotarod, beam balance and beam walking tasks have not been widely used and when they have, again often small, transient effects are seen both comparing SAH to sham-operated animals and in detecting treatment effects. Neurobehavioral tests have only recently been reported in rats and the results are conflicting with one group showing robust effects and another only minor differences [38,39]. Different models were used and the results were markedly different.

There are other neurobehavioral tests that assess neurobehavior in rodents, dogs and other species [41,58,109]. Neurobehavioral deficits in humans with SAH have been reported [110-112] but they need to be reviewed in detail so tests that assess these deficits in animal models can be used. The purpose would first be to be able to use animal models to predict whether a treatment would work in humans. This is a problem now in stroke research because a drug is tested in rodents and determined to decrease infarct size or neuronal damage and then it is tested in humans and has no effect. The trials in humans are costly and time-consuming so a better method to correlate outcome in animals and humans might facilitate testing of the most potentially efficacious treatments in humans. Second, better understanding of the pathogenesis of the disease, such as SAH in this case, hopefully would be forthcoming. For example, it is still unclear why neurobehavior is affected after SAH and whether this is due to increased intracranial pressure, SAH or a combination.

## Authors' contributions

HJ reviewed the literature and wrote the first draft of the paper. JA revised the first draft and searched the literature. MS, AT, XS and GC read the manuscript, searched the literature and contributed to the second draft. RLM formulated the idea, searched the literature, basically rewrote the second draft, formatted the manuscript and submitted it.
